# Identification of a New QTL Region on Mouse Chromosome 1 Responsible for Male Hypofertility: Phenotype Characterization and Candidate Genes

**DOI:** 10.3390/ijms21228506

**Published:** 2020-11-12

**Authors:** Magalie Vatin, Marie-Sophie Girault, Virginie Firlej, Carmen Marchiol, Côme Ialy-Radio, Xavier Montagutelli, Daniel Vaiman, Sandrine Barbaux, Ahmed Ziyyat

**Affiliations:** 1 Institut Cochin, Université de Paris, INSERM, CNRS, F-75014 Paris, France; magalie.inamo-vatin@univ-antilles.fr (M.V.); marie-sophie.girault@inserm.fr (M.-S.G.); virginie.firlej@u-pec.fr (V.F.); carmen.marchiol@inserm.fr (C.M.); come.ialy-radio@inserm.fr (C.I.-R.); daniel.vaiman@inserm.fr (D.V.); sandrine.barbaux@inserm.fr (S.B.); 2 Mouse Genetics Laboratory, Institut Pasteur, F-75015 Paris, France; xavier.montagutelli@pasteur.fr; 3 Service d’histologie, d’embryologie, Biologie de la Reproduction, AP-HP, Hôpital Cochin, F-75014 Paris, France

**Keywords:** infertility, spermatogenesis, teratozoospermia, globozoospermia, IRCS

## Abstract

Male fertility disorders often have their origin in disturbed spermatogenesis, which can be induced by genetic factors. In this study, we used interspecific recombinant congenic mouse strains (IRCS) to identify genes responsible for male infertility. Using ultrasonography, in vivo and in vitro fertilization (IVF) and electron microscopy, the phenotyping of several IRCS carrying mouse chromosome 1 segments of *Mus spretus* origin revealed a decrease in the ability of sperm to fertilize. This teratozoospermia included the abnormal anchoring of the acrosome to the nucleus and a persistence of residual bodies at the level of epididymal sperm midpiece. We identified a quantitative trait locus (QTL) responsible for these phenotypes and we have proposed a short list of candidate genes specifically expressed in spermatids. The future functional validation of candidate genes should allow the identification of new genes and mechanisms involved in male infertility.

## 1. Introduction

In approximately half of the 15% of couples that suffer from sterility, the cause is ascribed to male infertility, which encompasses a wide variety of syndromes. In more than half of infertile men, the cause is classified as idiopathic and may be due to genetic factors. Genetic defects leading to male infertility often affect spermatogenesis or sperm function [1].

Spermatogenesis is a biological process composed of a series of highly complex cellular events. It can be broadly divided into five discrete events: (1) renewal of spermatogonial stem cells and spermatogonia via mitosis, (2) proliferation (via mitosis) and differentiation of spermatogonia, (3) meiosis, (4) spermiogenesis (transformation of round spermatids into elongated spermatids and spermatozoa) and (5) spermiation, the release of sperm from the epithelium into the tubule lumen. All steps are highly regulated and dysfunctions in this key physiological process can result in infertility. Regulation of this complex process depends on the cooperation of many genes, which are expressed at these different steps [2]. Problems during spermatogenesis are most often reflected in a no or low production of spermatozoa and are described by routine semen analysis as azoospermia, oligozoospermia, teratozoospermia, asthenozoospermia or a combination of the last three (oligoasthenoteratozoospermia). Teratozoospermia is defined by morphologic sperm abnormalities. Globozoospermia is a rare and severe form of teratozoospermia characterized by round-headed spermatozoa lacking an acrosome, an important and specific organelle that plays a crucial role during fertilization. During spermiogenesis, diverse cellular and molecular processes allow sperm head formation and organization. Mutant models have improved the understanding of the etiology of teratozoospermia and clarified some of the mechanisms involved in sperm head formation and organization [3].

Identification of genes that play a crucial role in spermatogenesis is mainly based on observations in rodents. In particular, the mouse model appears to be an excellent model to study human infertility due to the conservation of the great majority of genes and processes involved in sperm production [4]. In order to identify genetic causes of infertility, we used the interspecific recombinant congenic mouse strains (IRCS) panel developed at the Institut Pasteur (Paris, France) [5]. The IRCS model harbors about 2% of *Mus spretus* (SEG/Pas) genome at a specific and known location inside a *Mus musculus domesticus* (C57BL/6J strain) background. The high polymorphism between *spretus* and *musculus* makes it possible to identify the quantitative trait locus (QTL) regions responsible for phenotypic variation in a given IRCS strain compared to the reference strain, C57BL/6J (B6) [5,6,7,8,9]. The phenotyping of the Rc3 strain, a congenic strain derived from the BcG-66H IRCS, led us to identify spermatogenesis defects such as acrosomal or flagellar anomalies that are absent in the B6 reference strain. The combination of phenotypic and fine mapping approaches allowed us to map the Mafq1 (male fertility QTL chromosome 1) locus on a unique *spretus* fragment on mouse chromosome 1 and to identify a short list of candidate genes responsible for the spermatogenesis defects and male hypofertility of the Rc3 strain.

## 2. Results

### 2.1. In Vivo Phenotyping

**Figure 1:**
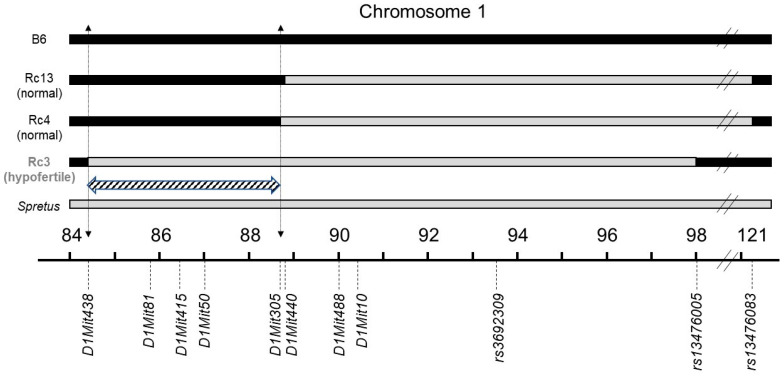
Genotypes of the different recombinant strains (Rc) and the parental strains (*spretus* and B6). Recombinant strains (Rc) were generated at the Institut Pasteur (Paris) from the 66HMMU1 strain by recombination events inside the MMU1 *spretus* segment. Black regions correspond to B6 background, grey regions to *spretus* fragments and the minimal *spretus* region responsible for the phenotype of interest is highlighted in hatched on chromosome 1. Marker positions are given in mega base pairs (Mb). Genome version GRCm38.p6.

We have previously presented the generation of the subcongenic substrains (named Rc) from the 66HMMU1 strain by recombination events inside the MMU1 *spretus* segment [9]. Each of these substrains differ from B6 (control) due to the presence of a unique *spretus* fragment on the chromosome 1 in their genome that overlaps with those of the other substrains (Figure 1).

**Figure 2:**
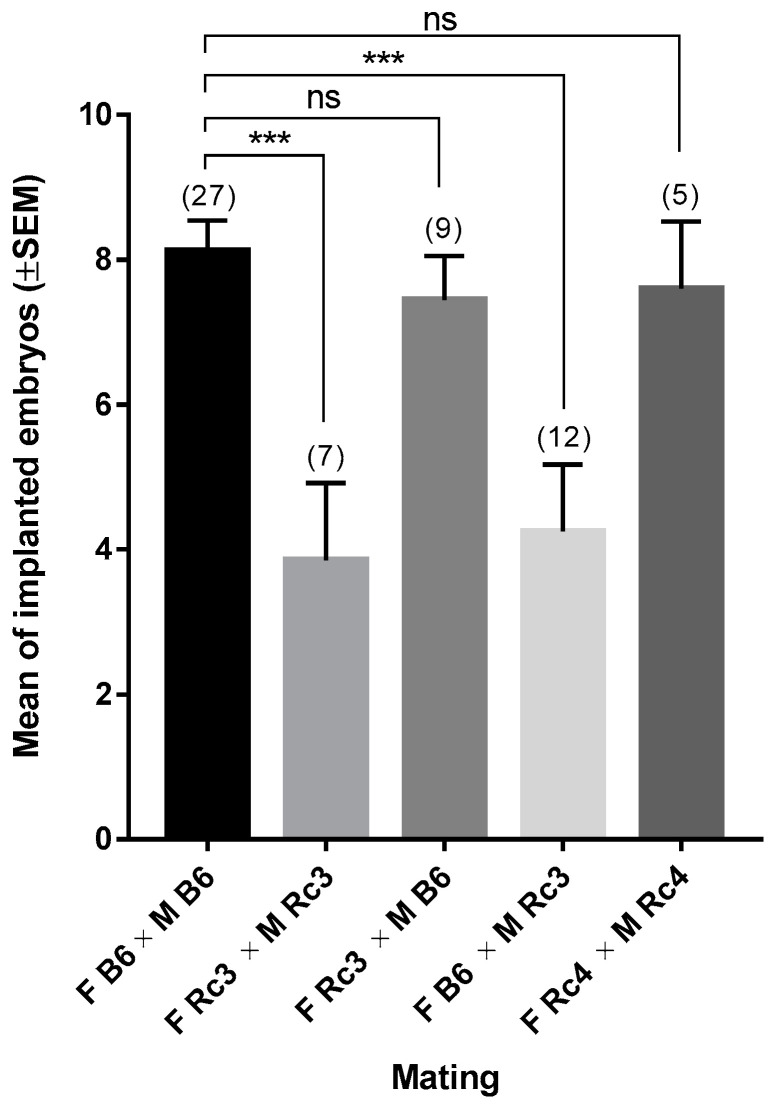
Ultrasonographic examination of the implantation in different crosses using mice from Rc3, Rc4 and B6 strains: Different crosses were performed using males (M) and females (F) from Rc3, Rc4 and B6 strains. The results are presented as the mean (±SEM) of implanted embryos. ( ): number of gestations. ns: non significant. ***: *p* < 0.001.

Here we present the phenotyping of the Rc3 substrain compared to the B6 strain, by using an in vivo ultrasonic method to evaluate the implantation rate at E7.5 and E9.5. For this, we carried out inbred crosses between males and females from each substrain and determined the mean of implanted embryos. We observed a significant reduction in the mean number of implanted embryos in the Rc3 × Rc3 crosses compared to B6 × B6 controls. To know whether the defect observed in Rc3 orginated from the female or the male side, we performed two reciprocal F1 crosses: Rc3 males with B6 females and B6 males with Rc3 females. The number of implanted embryos was reduced only in the crosses involving Rc3 males (*p* < 0.001) and we concluded that the defect was due to the male. The Rc4 substrain, which carries a partially overlapping *spretus* chromosomal fragment did not show a hypofertility phenotype (Figure 2).

### 2.2. Phenotype Characterization

**Figure 3:**
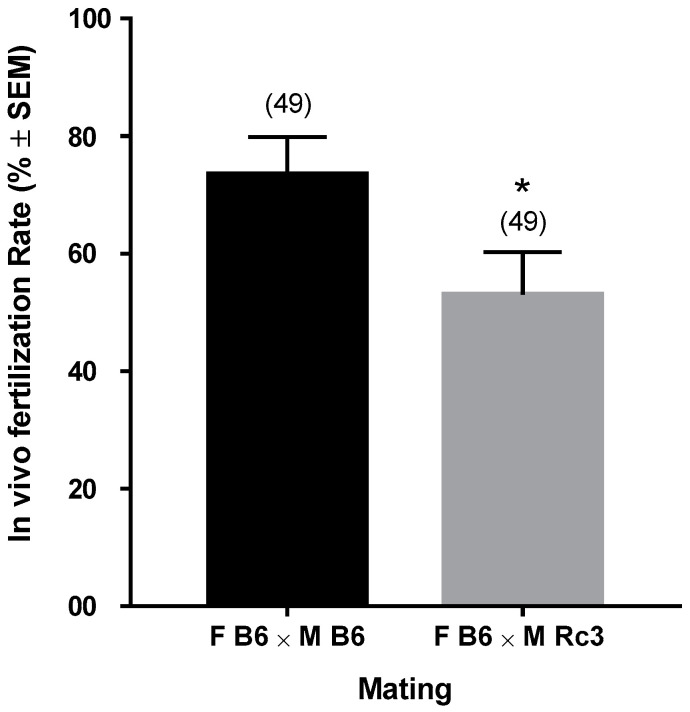
Evaluation of in vivo fertilization: B6 females were mated with B6 or Rc3 males to evaluate their respective capacity to fertilize oocytes in vivo. The mating was performed on the night and the next day, oocytes from oviducts of females with a positive vaginal plug were collected. The fertilization was assessed using 4′,6-diamidino-2-phenylindole (DAPI) fluorescent staining. The results are presented as the mean of the fertilization rate (%) ± SEM using t-test. * *p* = 0.036. F: female, M: male. ( ) number of oocytes.

To identify the origin of the male defect(s), we focused on mating between Rc3 males and B6 females. The next day, to verify in vivo fertilization, we collected and counted the number of fertilized oocytes from the ampullae of the females presenting with a vaginal plug. We compared the results from the B6 × Rc3 with those from the B6 × B6 controls and we observed a significant (*p* = 0.036) reduction in the fertilization rate with Rc3 males ranging from 73 ± 6% for the B6 control to 53 ± 7% for the Rc3 substrain (Figure 3).

**Figure 4:**
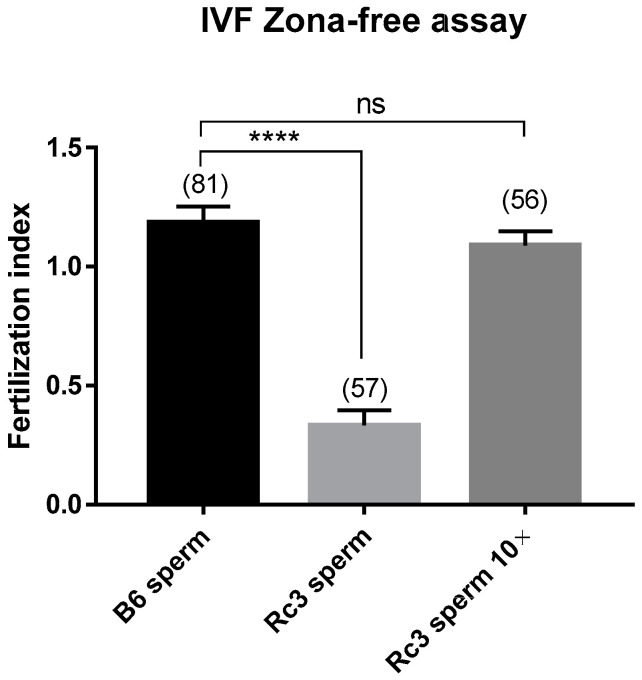
Evaluation of in vitro fertilization (IVF). The fertilization index (or mean ± SEM of sperm number fused by egg) was assessed by zona-free IVF assay at 10^5^ (B6 and Rc3 sperm) or 10^6^ (Rc3 sperm 10×) sperm per mL. Experiments were repeated at least three times. ( ): number of oocytes. ns: non significant. ****: *p* < 0.0001.

We also performed in vitro fertilization (IVF) using B6 oocytes and Rc3 versus B6 epididymal sperm. We observed a significant reduction of Rc3 (B6 oocytes and Rc3 epididymal sperm) fertilizing ability with a Fertilization Index (FI: mean of sperm number fused by egg) of 0.33 ± 0.06, compared to B6 controls (B6 oocytes and B6 epididymal sperm) with a FI of 1.18 ± 0.06 (*p* < 0.0001). Interestingly, when we increased the concentration of Rc3 sperm (10 times), the FI was brought back to normal values (FI = 1.08 ± 0.05, *p* = 0.47) (Figure 4).

### 2.3. Characterization of the Sperm Defect of the Rc3 Males

**Figure 5:**
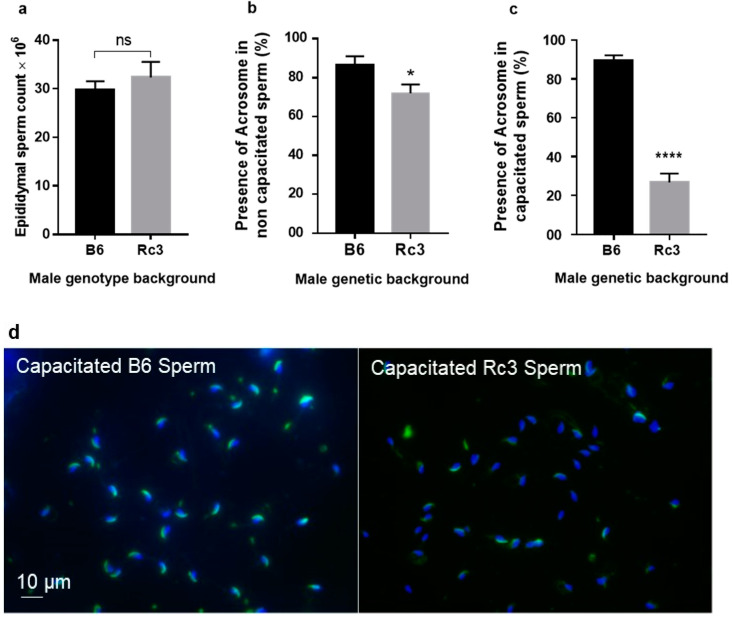
(**a**). Evaluation of epididymal sperm count on B6 and Rc3 using Malassez slide. Estimation of the percentage of the presence of acrosome on non-capacitated (**b**) and capacitated (**c**) B6 and Rc3 epididymal sperm. (**d**) As shown in these illustrative slides, for this analysis, nuclei were stained with DAPI (blue) and the acrosomal region with PSA-FITC (green). ns: non significant, * *p* = 0.03, **** *p* < 0.0001.

To identify the origin of this sperm dysfunction, we first counted the sperm of B6 and Rc3 males used for IVF assays and we did not observe any significant difference between the two groups (29.67 ± 1.85 × 10^6^ (n = 3) for the B6 males and 32.33 ± 3.18 × 10^6^ (n = 3) for the Rc3 males) (Figure 5a, *p* = 0.5).

After an inconclusive morphological observation under phase-contrast microscopy, Rc3 sperm were observed and compared to B6 sperm using fluorescence microscopy with PSA-FITC (Pisum sativum agglutinin conjugated to fluorescein) labelling to detect the presence of sperm acrosome and DAPI (4′,6-diamidino-2-phenylindole) for nucleus. A slight difference (*p* = 0.03) in the presence of sperm acrosome was observed on non-capacitated freshly recovered epididymal sperm, in which the acrosome was present in 86.4 ± 4.5% of B6 sperm and in 71.8 ± 4.6% of Rc3 sperm (Figure 5b). After 90 min of capacitation in Ferticult medium supplemented with 3% Bovine Serum Albumin (BSA) at 37 °C under 5% CO_2_, this percentage remained approximately the same for B6 sperm (89.4 ± 2.6%), while in sperm from Rc3 males this percentage dropped drastically to only 26.8 ± 4.5% (*p* < 0.0001, Figure 5c). These results likely reflect a defect at the acrosome level, which leads to early loss of the acrosome during capacitation by spontaneous acrosome reaction (sAR).

These results were completed by observing the sperm motility under a microscope, as carried out during IVF experiments. While B6 sperm showed a total mobility exceeding 50% on average, those of Rc3 rarely exceeded 20%. When we looked at the fraction of sperm that showed progressive motility, this percentage dropped from 20% to 25% for B6 sperm to 10% at best for Rc3 sperm.

**Figure 6:**
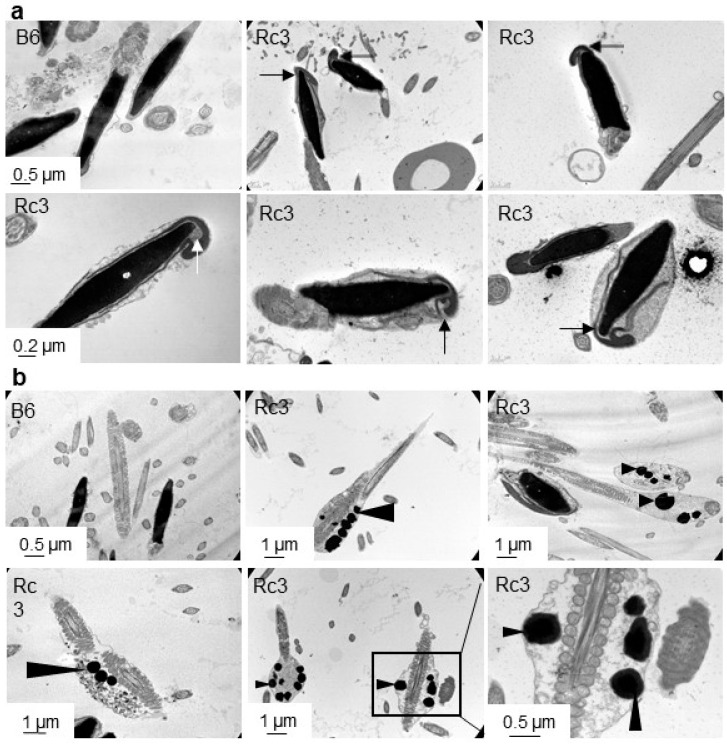
Observation of B6 and Rc3 sperm heads (**a**) and flagella (**b**) using electron microscopy. No abnormality was observed on B6 sperm where nuclear and inner acrosomal membranes seem joined, whereas the acrosome seems abnormally attached to the nucleus in approximately 50% of the sperm head of Rc3 males (**a**, arrows), in addition to the presence of lipid droplets (**b**, arrowheads) in the residual bodies at the level of the midpiece in approximately 15% of the sperm.

Alterations in sperm could explain the high rate of spontaneous acrosomal reaction that occurred during capacitation. In order to investigate whether discrete alterations exist at the ultrastructural level, a second observation of Rc3 and B6 sperm was performed using electron microscopy (EM). A slight acrosome defect consisting of a detachment between the anterior part of the acrosome and the nucleus was observed in about half of Rc3 sperm (Figure 6a). This acrosomal defect was not seen in B6 control sperm. EM also revealed anomalies of the flagella (Figure 6b). Indeed in Rc3 sperm, residual bodies at the flagella, also not found on B6 sperm, were observed in 15 to 20% of sperm. These residual bodies contain large lipid droplets that likely reflect a metabolic dysfunction and/or spermiation defect.

### 2.4. QTL Fine Mapping

The Rc3 substrain differs from the B6 strain by a *spretus* fragment of about 14 Mb on the MMU1 chromosome delimited by *D1Mit438* and *rs13476005* markers (see Figure 1). This *spretus* region contains gene(s) responsible for the observed phenotype. We undertook to decrease the size of the fragment.

Since the Rc4 strain does not show the hypofertility phenotype (Figure 1 and Figure 2), we excluded the *spretus* region of 4 Mb between the two markers, *D1Mit305* and *rs13476005* that are shared by Rc3 and Rc4. Therefore, we defined a QTL region of about 4.2 Mb on chromosome 1 between *D1Mit438* and *D1Mit305* (hatched zone in Figure 1) as responsible for male hypofertility. We named this QTL *Mafq1* (Male fertility QTL chromosome 1).

### 2.5. Identification of Candidate Genes in the QTL Region Responsible for Rc3 Sperm Phenotype

We explored the *Mafq1* interval to identify candidate genes whose presence in the *spretus* haplotype within a B6 background could be responsible for the hypofertility phenotype. Table S1 compiles all sequences described in different databases as protein coding genes (n = 71), processed transcripts (n = 37), pseudogenes (n = 50) and non coding RNAs (n = 48). We also documented their expression profile, when available, at the RNA and protein level, in order to focus on genes expressed in the testis and preferably at the spermatid stage to explain the dysfunction of the acrosome. Some genes have already been invalidated in mouse models (n = 28), and although some of these knockout (KO) models expressed phenotypes that are overall related to reproduction, notably, none of them could recapitulate the particular features observed in the Rc3 mouse. The supposed or validated function of the protein has also been documented in the literature. Finally, we counted the single nucleotide polymorphisms (SNPs) with putative functional impact between *spretus* and B6, particularly in the open reading frames where they could be responsible for structural changes affecting protein interactions. Therefore, we applied these filters to select the most relevant candidates genes. We identified 8 genes highly expressed in the testis: *Trip12*, *Fbxo36*, *Spata3*, *Tex44*, *Pde6d*, *Efhd1*, *Dnajb3* and *Mroh2a*, of which two are exclusively expressed in the testis (*Spata3* and *Tex44*).

**Figure 7:**
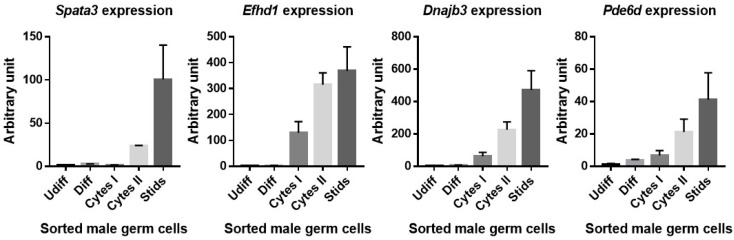
Expression level of four genes during the different stages of spermatogenesis. To determine the stage of spermatogenesis in which these four genes are expressed, the different male germ cell populations were separated by flow cytometry and the expression levels were measured by qRT-PCR. Undifferentiated (Udiff), differentiated (Diff) spermatogonias, primary (Cytes I) and secondary (Cytes II) spermatocytes, spermatids (Stids).

Because the differences in the expression level (mRNA) depended on the databases, we performed qRT-PCR experiments in order to analyze the expression of several candidate genes in the testis. The different cellular populations of the testis were sorted by flow cytometry, and the expression level of *Spata3*, *Dnajb3, Pde6d* and *Efhd1* was evaluated (Figure 7). While three of them appeared to be expressed at different stages of spermatogenesis, *Spata3* was exclusively expressed at the spermatid stage.

## 3. Discussion

In previous studies, we have shown that IRCS mice can provide novel phenotypes of interest and that they have the power to identify the responsible gene [9,10,11,12]. As an example, we have proposed *Fidgetin-like1* (on chromosome 13) as a strong candidate for the dynamic impairment of male meiosis, which leads to reduced testis weight in mice [8]. This gene has been described as pivotal for meiotic recombination [13]. This approach allows the identification of new loci and genes that can be important for gametogenesis and whose study can improve our knowledge about infertility disorders.

Similarly, here, using these IRCS lines, we have shown an association between a QTL on chromosome 1 (*Mafq1)* present in the IRCS-derived Rc3 subcongenic strain and male hypofertility. For this, we first used the ultrasonic method to estimate the number of implanted embryos in reciprocal crosses involving B6 and Rc3 females mated with either Rc3 or B6 males. The number of implantation sites was significantly lower in both B6 and Rc3 females when mated with Rc3 males, although we did not observe any difference in terms of embryonic resorption. This led us to assess the fertilizing ability of Rc3 sperm. Indeed, in vivo as in vitro, the fertilization rate was found to be lower with the sperm obtained from Rc3 males. We did not observe any obvious morphological alterations under light microscopy but found reduced mobility in Rc3 sperm; thus, we increased the sperm concentration of Rc3 by 10 during IVF. This made it possible to restore the fertilization index to the same level with B6 sperm. The decrease in fertilization ability observed in vitro was greater than that observed in vivo. This difference is probably explained by the optimal conditions and high efficacy of in vivo fertilization compared to in vitro fertilization [14]. The second explanation is that the sperm make a long journey in the female genital tract. The sperm motility is one of the elements contributing to the success of this journey. As a result, a form of selection takes place and sperm with significantly reduced motility do not reach the oviduct. Conversely, in vitro, sperm are directly deposited in contact with oocytes and even those with reduced motility participate to the fertilization. This could further reduce the fertilization rate. Therefore, IVF better reveals minor functional alterations, as seen in our other examples regarding sperm from *Spaca6* heterozygous mutant males [15].

We then looked for the presence of the acrosome. While this was similar on non-capacitated sperm, the acrosome seemed to be absent more often after capacitation in Rc3 sperm, reflecting an early sAR on Rc3 sperm and the potential fragility of the acrosome. Early sAR may not enable the fertilization process to proceed but the timing of the acrosome reaction (AR) seems to be flexible [16]. The spontaneous acrosomal reaction is a physiological phenomenon, but its frequency increases in the Rc3 strain [17,18]. In humans, sperm with high proportion of sAR result in poor success in IVF [19]. Via its ability to prevent early sAR before reaching the female genital tract, Paraoxonase 1 activity seems to have a positive effect on fertility [20]. In mice, this question remains debatable. For example, the disruption of mouse *CD46* causes an accelerated sAR, but also the facilitation of fertilizing ability of males [21]. Supporting this idea, Sebkova et al. showed that the relocation of Izumo1 takes place normally even after sAR [18]. This apparent contradiction could probably be explained by the exact timing and magnitude of this early acrosomal reaction. These questions could be addressed by invalidating candidate genes.

The electron microscopy analysis finally made it possible to specify the defect in Rc3 sperm by highlighting a detachment of the acrosome that does not adhere correctly to the nucleus. This indicates that the protein involved in this phenotype could be localized at the level of the inner membrane of the acrosome, of the nuclear membrane, or even between the two at the level of the acroplaxome. If not, this protein could be localized elsewhere but have an indirect action via a regulatory role. Such a phenotype has already been observed in *Hipk4* (homeodomain-interacting protein kinase 4) KO mice demonstrating that this gene is essential for murine spermiogenesis as a regulator of the shape of the sperm head [22], or in germ cell-specific *Sirt1* KO infertile mice where disrupted spermiogenesis caused defects in acrosome biogenesis, which resulted in a phenotype similar to that observed in human globozoospermia [23]. In addition, the absence of DPY19L2, an inner nuclear membrane protein, causes globozoospermia in human and in mice by preventing the anchoring of the acrosome to the nucleus [24,25]. The phenotype that we describe here suggests an abnormal organization of the acrosome. Admittedly, the observed phenotype is not as drastic as those mentioned above, which might suggest that the genetic defect in the Rc3 strain, probably resulting from the presence of a protein with altered function, could be different from the one observed in the complete absence of the protein as in the case of KO mice. However, this difference could be sufficient to prevent, at least partially, normal protein interactions, thus reinforcing the idea of the “transcriptomic shock” resulting from the brutal contact between two divergent genomes [6]. This morphological abnormality seems to characterize a teratozoospermia, like partial globozoospermia.

The second phenotype revealed by electron microscopy is the presence of residual cytoplasm that contains numerous lipid droplets on a fraction of Rc3 sperm. This phenotype reflects some defects in spermiation. Because the epidydimal sperm concentration in Rc3 strain was similar to that of the controls, we can conclude that the production and disengagement of spermatids, and their release towards the lumen of the seminiferous tubules was normal. Nevertheless, about 20% of Rc3 sperm still present residual bodies, which suggests a defective phagocytosis process. This phenotype could have its origin in a dysfunction either at the spermatids level or at the Sertoli cells level and could also explain the persistence of lipid droplets in the residual bodies. Indeed, the phagocytosis of residual bodies is associated with a peak in the number of lipid droplets at the base of the Sertoli cell, as observed in various species [26]. Alternatively, the presence of these lipid droplets in epidydimal sperm could also reflect a metabolic dysfunction.

At this stage, it is not possible to know whether one and the same protein is responsible for the two distinct observed phenotypes. Further investigations are needed. These will probably require invalidating one or more genes present in the *Mafq1* interval. We hypothesize that one or more genes from the interval have an epistatic interaction with one or more genes outside of the interval; in the Rc3 strain, the *spretus* allele(s) fail to interact properly with the B6 allele(s). This relation could be the participation of heterodimers to a proteic complex or ligand-receptor binding for example. One of these candidate genes could be related to the *Hipk4*, *Sirt1* or *Dpy9l2* genes, whose invalidation affects acrosome biogenesis in mice or humans. The production and fine genetic characterization of partially overlapping strains defined a genetic interval associated with the above-described phenotypes.

The number of genes is important in this interval (71 coding genes); therefore, we then applied several filters in order to prioritize those that are more likely to cause the phenotypes observed during our study.

According to the expression database that was consulted, 22 genes out of a total of 71 are reported to be expressed in the testis (see Table S1). The inactivation of 14 of them did not result in spermatogenesis defects (for references, see Table S1). Of these 14 genes, the case of *Dnajb3* is questionable. Indeed, this gene was initially described as exclusively expressed in the testis and particularly at the spermatid stage [27,28] in the developing acrosomal vesicle and sperm centriolar region [29]. However, other studies found its expression in other tissues [30,31,32,33]. Its role seemed to be related to proteasome and protein quality control [34] but also to insulin signaling, glucose uptake and oxidative stress [30,35]. Although only two coding SNPs differ between B6 and *spretus*, the *spretus* allele of one of them is predicted to be deleterious on protein structure (SIFT, Ensembl (www.ensembl.org)) (Table S1). Finally, a KO strain available from the Jackson Laboratory (https://www.mousephenotype.org/data/genes/MGI:1306822) showed no reproductive phenotype. These latest data seem to downplay the role of *Dnajb3* in spermatogenesis, either because its participation in this process is not essential or because its function is compensated for by another gene in KO models.

Among the genes remaining in the short list and for which the KO model does not exist, *Armc9* gene mutations have been described in human Joubert syndrome, but the variety of phenotypes does not include a phenotype related to reproduction. In addition, Van De Weghe et al. found that CRISPR/Cas9-mediated KO of *armc9* in zebrafish resulted in curved body shape, retinal dystrophy, coloboma, reduced cilia number in ventricles, and shortened cilia in photoreceptor outer segments [36]. *Cops7b* and *Chrnd* are not expressed in mouse testis but their expression is described in human testis. Mutations of *CHRND* gene in humans have been reported to be associated with congenital myasthenic syndrome [37,38,39]. *Efhd1* is expressed in testis but also in ovary, kidney and placenta (Table S1). *Mroh2a* shows ubiquitous expression but with a higher level of expression in the testis while *Hjurp* is not expressed in mouse testis. The testicular expression of the *Glrp1* gene is described in one database but not in another and no orthologous gene is described in humans.

The filters that we applied make it possible to exclude some genes such as those that are not expressed in the testis, those for which the KO does not show a spermatogenesis phenotype and even those without significant SNPs. These filters make it possible to highlight two genes that are probably involved in the observed phenotype. These are *Spata3* and *Tex44*, which present an expression profile restricted to the testis, and particularly in the spermatid. Our verification by qRT-PCR indicated the post-meiotic expression (at the spermatid stage) of *Spata3,* which confirmed the data found in the expression databases. The expression profile at the protein level given by Protein Atlas indicates their presence only at the spermatid stage in human testis. These two genes have not been studied in the literature, and sequence analysis does not help to relate them to known protein families and to suggest a particular function. Although the name “*Spata*”, meaning “spermatogenesis associated” is common to several dozen genes, no particular functions or domains link these genes. As for *Tex44*, *Spata3* was identified as a gene very recently. Nevertheless, they both accumulate SNPs in coding sequences that could modulate protein structure and/or function and interactions with other partners expressed in a B6 version in the IRCS. Therefore, they appear as the best candidates.

## 4. Materials and Methods

### 4.1. Ethics Statement

All animal experiments were performed in accordance with national guidelines for the care and use of laboratory animals. Authorizations were obtained from local (C2EA-34, Comité d’éthique en matière d’expérimentation animale Paris Descartes) and governmental ethical review committees via APAFIS Application (Autorisation de projet utilisant des animaux à des fins scientifiques), Authorization APAFIS #14124-2017072510448522 v26, A. Ziyyat (2018–2023).

### 4.2. Animals

The generation of the recombinant substrains from BcG-66H and 66HMMU1 at the Institut Pasteur (Paris) has been previously reported [5,9]. After weaning, 4-week-old mice were maintained in an animal facility at the Cochin Institute (Paris) at normal temperature (21–23 °C) and 14 h light/10 h dark photoperiods with free access to water and food. For all experiments, animals were sacrificed by cervical dislocation.

### 4.3. Phenotyping by High Frequency Ultrasonography Evaluation of in Vivo Fertilization

To evaluate the implantation rate, mice from B6 (reference strain) and Rc strains were crossed and phenotyped at the small animal imaging facility of the Cochin Institute using high frequency ultrasonography (VEVO 770, Visulasonics, Toronto, Canada). Each female was used one time to collect phenotypic data from the primo-gestation. Briefly, a chemical hair remover was used to eliminate abdominal hair. Ultrasonographic contact gel was used to ensure contact between the skin surface and the transducer. Body temperature, electrocardiographic and respiratory profiles were monitored using the device’s integrated heating pad and monitoring device (THM150, Indus Instruments, Webster, TX, USA). The implantation rate was determined early in the gestation, at E7.5 and E9.5. At these stages, the small size of the embryos permits a fluent count and resorbed embryos are also visible.

### 4.4. Evaluation of in Vivo Fertilization

B6 females were mated with Rc3 or B6 males at the night (one couple per cage) and the next day females with a vaginal plug were isolated. Oocytes were collected from ampullae of oviducts and freed from the cumulus cells by brief incubation at 37 °C with hyaluronidase (Sigma, St. Louis, MO, USA) in M2 medium (Sigma). Oocytes were rinsed and kept in M2 medium in a humidified 5% CO_2_ atmosphere at 37 °C before mounting. The number of fertilized oocytes was evaluated using DAPI immune-fluorescent staining to visualize the DNA.

### 4.5. In Vitro Fertilization Assays

Oocytes Preparation: Five week-old B6 females were injected with 5 IU of PMSG (pregnant mare’s serum gonadotrophin; Intervet, France) followed by 5 IU of hCG (human chorionic gonadotrophin; Intervet) 48 h later. After super-ovulation, cumulus-oocyte complexes were collected from ampullae of oviducts about 14 h after hCG injection. Oocytes were freed from the cumulus cells by 3–5 min of incubation at 37 °C with hyaluronidase (Sigma) in M2 medium (Sigma). Oocytes were rinsed and kept in Ferticult medium (FertiPro, Belgium) at 37 °C under 5% CO_2_ atmosphere under mineral oil (Sigma). Zona pellucida was then dissolved with acidic Tyrode’s (AT) solution (pH 2.5, Sigma) under visual monitoring. The zona-free eggs were rapidly washed in medium and kept at 37 °C under 5% CO_2_ atmosphere for 2 to 3 h to recover their fertilizability.

Sperm preparation: Mouse spermatozoa were collected from the caudae epididymis of 8–13-week-old B6 and Rc3 males and capacitated at 37 °C under 5% CO_2_ for 90 min in a 500 µl drop of Ferticult medium supplemented with 3% BSA (Sigma), under mineral oil.

In Vitro Fertilization: Zona-free eggs were inseminated with capacitated sperm for 3 h in a 100 µl drop of Ferticult 3% BSA medium at a final concentration of 10^5^/mL or 10^6^/mL when the aim was to try to recover fertilizing ability of Rc3 sperm by concentrating it ten times. Then, they were washed and directly mounted in Vectashield/DAPI (Vector laboratories, CA, USA) for microscopy observation. The oocytes were considered fertilized when they showed at least one fluorescent decondensed sperm head within their cytoplasm.

### 4.6. Assessment of Sperm Acrosome Reaction

Freshly recovered or capacitated sperm were washed in PBS containing 1% BSA, centrifuged at 300× *g* for 5 min and immediately fixed in 4% paraformaldehyde (Electron Microscopy Sciences, Hatfield, PA, USA) in PBS with 1% BSA at 4 °C for 1 h. In order to detect the sperm acrosomal status, after washing, the fixed spermatozoa were treated for 30 min in 95% ethanol at 4 °C, washed by centrifugation through PBS and stained with FITC-conjugated lectin PSA (25 μg/mL in PBS) for 10 min. Nuclei were stained with DAPI (blue). After repeated washing with double distilled water, a drop of sperm suspension was smeared on a slide, air-dried, and mounted with Vectashield. Detection was performed using a Nikon Eclipse E600 microscope Zeiss Axiophot epifluorescence microscope and images were digitally acquired with a camera (Coolpix 4500, Nikon).

### 4.7. Transmission Electron Microscopy Analysis of Sperm Cells

Mouse spermatozoa from three different males were prepared as described above (in vitro fertilization) and fixed by incubation in PBS supplemented with 2% glutaraldehyde (Grade I, Sigma) for 2 h at room temperature. Samples were washed twice in PBS and post-fixed by incubation with 1% osmium tetroxide (Electron Microscopy Sciences), after which they were dehydrated by immersion in a graded series of alcohol solutions and embedded in Epon resin (Polysciences Inc., Warrington, PA, USA). Ultra-thin sections (90 nm) were cut with a Reichert Ultracut S ultramicrotome (Reichert-Jung AG, Wien, Austria) and were then stained with uranyl acetate and lead citrate. Sections were analyzed with a JEOL 1011 microscope and digital images were acquired with a Gatan Erlangshen CCD camera and Digital Micrograph software. The integrity of sperm organelles was checked including the acrosome, nucleus, flagellum, etc. Acrosomes with a space between the nuclear membrane and the inner acrosomal membrane were considered abnormal. Acrosomes with membranes in close contact were considered normal. The presence of residual bodies, cytoplasmic leftovers, was recorded.

### 4.8. Digital and Bibliographic Tools Used to Shorten the List of Candidate Genes

Databases were searched for the presence of genic sequences including UCSC GenomeBrowser (genome.ucsc.edu), Ensembl (www.ensembl.org), and NCBI Gene (www.ncbi.nlm.nih.gov/gene). The nature of these sequences was reported as protein coding gene, processed transcript, pseudogene, or non coding RNA, although some discrepancies exist between databases. The RNA expression level was searched in the BioGPS (biogps.org) and NCBI Gene databases, in mouse and in human when available. Results from antibody labelling on human tissues were obtained from ProteinAtlas (proteinatlas.org). Data relative to mouse knock-out models and human diseases were collected from the Mouse Genome Informatics website (www.informatics.jax.org). Protein function was obtained from the Gene Ontology website (www.geneontology.org). SNPs were retrieved from the Mouse Genomes Project (www.sanger.ac.uk/sanger/Mouse_SnpViewer) and MGI databases. All analyses were done on the GRCm38 version of the mouse genome.

### 4.9. Suspension Cell Sorting

Testis cells from 2-month-old B6 mice were obtained as described previously [40]. The testis albuginea was removed and seminiferous tubules were dissociated using enzymatic digestion by collagenase type I at 100 u/mL for 15 min at 32 °C in HBSS supplemented with 20 mM HEPES pH 7.2, 1.2 mM MgSO_4_ 7H_2_O, 1.3 mM CaCl_2_ 2H_2_O, 6.6 mM sodium pyruvate, and 0.05% lactate. After an HBSS wash and centrifugation, the pelleted tubules were further incubated in cell dissociation buffer (Invitrogen) for 25 min at 32 °C. The resulting whole cell suspension was successively filtered through a 40 μm then 20 µm nylon mesh to remove cell clumps. After an HBSS wash, the cell pellet was resuspended in incubation buffer (HBSS supplemented with 20 mM HEPES pH 7.2, 1.2 mM MgSO_4_ 7H_2_O, 1.3 mM CaCl_2_ 2H_2_O, 6.6 mM sodium pyruvate, 0.05% lactate, glutamine and 1% fetal calf serum) and stained with Hoechst 33,342 (5 µg/mL) for 1 h at 32 °C in a water bath. Cells were then labeled with monoclonal antibodies (1 µg per 10^6^ cells) from BD Pharmingen: anti-c-Kit-biotin (2B8), and anti-α-6 integrin-PE (GoH3). The cell sorting was performed on ARIA (Becton Dickinson).

### 4.10. RNA Extraction and Quantitative RT-PCR

Total RNA was extracted using TRIzol Reagent (Invitrogen, Carlsbad, CA, USA) in accordance with the manufacturer’s instructions. After RNA preparation, total RNA was treated with DNase I (Invitrogen Life Technologies) for 10 min at room temperature followed by inactivation with EDTA (Sigma). Total RNA was reverse transcribed to obtain cDNA using M-MLV Reverse Transcriptase (Invitrogen, Carlsbad, CA, USA) following the manufacturer’s protocols. Quantitative PCR was carried out using the fast SYBR Green Master Mix (Applied Biosystems) and a real time PCR system (Light Cycler 1.5, Roche Diagnostics, Division Applied Sciences, Meylan, France) according to standard PCR conditions. To validate the primers used in qRT-PCR, four pairs of primers were tested for each candidate gene and reference gene. For quantitative calculations, values were normalized to mouse Cyclophiline A expression.

### 4.11. Statistical Analysis

Results are expressed as mean ±SEM of at least three independent experiments. For statistical analysis, one-way ANOVA multiple comparisons test or t-Test were performed using GraphPad Prism version 7.00 for Windows, (GraphPad Software, La Jolla California USA). Differences were considered statistically significant when *p* < 0.05.

## Supplementary Materials

Supplementary Materials can be found at https://www.mdpi.com/1422-0067/21/22/8506/s1.

## Abbreviations

DAPI	4′,6-diamidino-2-phenylindole
sAR	Spontaneous acrosome reaction
AR	Acrosome reaction
QTL	Quantitative trait locus
IRCS	Recombinant interspecific congenic strain
IVF	In vitro fertilization
KO	Knockout
*Mafq1*	Male fertility QTL chromosome 1
