# Supplementary material for: Identification of a New QTL Region on Mouse Chromosome 1 Responsible for Male Hypofertility: Phenotype Characterization and Candidate Genes
Source: Int J Mol Sci. 2020 Nov 12;21(22):8506. doi: 10.3390/ijms21228506 (PMC7697627; doi:10.3390/ijms21228506)
Supplement: Supplementary file 1 [file ijms-21-08506-s001.zip › ijms-994581-supplementary References cited in Table S1 pending conversion.docx]

**References cited in Table S1**

1. Tohgo, A.; Eiraku, M.; Miyazaki, T.; Miura, E.; Kawaguchi, S.Y.; Nishi, M.; Watanabe, M.; Hirano, T.; Kengaku, M.; Takeshima, H. Impaired cerebellar functions in mutant mice lacking DNER. *Mol Cell Neurosci* **2006**, *31*, 326-333, doi:10.1016/j.mcn.2005.10.003.

2. Kajiro, M.; Tsuchiya, M.; Kawabe, Y.; Furumai, R.; Iwasaki, N.; Hayashi, Y.; Katano, M.; Nakajima, Y.; Goto, N.; Watanabe, T., et al. The E3 ubiquitin ligase activity of Trip12 is essential for mouse embryogenesis. *PLoS One* **2011**, *6*, e25871, doi:10.1371/journal.pone.0025871.

3. Dickinson, M.E.; Flenniken, A.M.; Ji, X.; Teboul, L.; Wong, M.D.; White, J.K.; Meehan, T.F.; Weninger, W.J.; Westerberg, H.; Adissu, H., et al. High-throughput discovery of novel developmental phenotypes. *Nature* **2016**, *537*, 508-514, doi:10.1038/nature19356.

4. Whyte, L.S.; Ryberg, E.; Sims, N.A.; Ridge, S.A.; Mackie, K.; Greasley, P.J.; Ross, R.A.; Rogers, M.J. The putative cannabinoid receptor GPR55 affects osteoclast function in vitro and bone mass in vivo. *Proc Natl Acad Sci U S A* **2009**, *106*, 16511-16516, doi:10.1073/pnas.0902743106.

5. Nebigil, C.G.; Choi, D.S.; Dierich, A.; Hickel, P.; Le Meur, M.; Messaddeq, N.; Launay, J.M.; Maroteaux, L. Serotonin 2B receptor is required for heart development. *Proc Natl Acad Sci U S A* **2000**, *97*, 9508-9513, doi:10.1073/pnas.97.17.9508.

6. Littlechild, S.L.; Young, R.D.; Caterson, B.; Yoshida, H.; Yamazaki, M.; Sakimura, K.; Quantock, A.J.; Akama, T.O. Keratan Sulfate Phenotype in the beta-1,3-N-Acetylglucosaminyltransferase-7-Null Mouse Cornea. *Invest Ophthalmol Vis Sci* **2018**, *59*, 1641-1651, doi:10.1167/iovs.17-22716.

7. Prendergast, C.E.; Morton, M.F.; Figueroa, K.W.; Wu, X.; Shankley, N.P. Species-dependent smooth muscle contraction to Neuromedin U and determination of the receptor subtypes mediating contraction using NMU1 receptor knockout mice. *Br J Pharmacol* **2006**, *147*, 886-896, doi:10.1038/sj.bjp.0706677.

8. Ueda, H.; Sasaki, K.; Halder, S.K.; Deguchi, Y.; Takao, K.; Miyakawa, T.; Tajima, A. Prothymosin alpha-deficiency enhances anxiety-like behaviors and impairs learning/memory functions and neurogenesis. *J Neurochem* **2017**, *141*, 124-136, doi:10.1111/jnc.13963.

9. Zhang, H.; Li, S.; Doan, T.; Rieke, F.; Detwiler, P.B.; Frederick, J.M.; Baehr, W. Deletion of PrBP/delta impedes transport of GRK1 and PDE6 catalytic subunits to photoreceptor outer segments. *Proc Natl Acad Sci U S A* **2007**, *104*, 8857-8862, doi:10.1073/pnas.0701681104.

10. Hunter, R.W.; Liu, Y.; Manjunath, H.; Acharya, A.; Jones, B.T.; Zhang, H.; Chen, B.; Ramalingam, H.; Hammer, R.E.; Xie, Y., et al. Loss of Dis3l2 partially phenocopies Perlman syndrome in mice and results in up-regulation of Igf2 in nephron progenitor cells. *Genes Dev* **2018**, *32*, 903-908, doi:10.1101/gad.315804.118.

11. Narisawa, S.; Frohlander, N.; Millan, J.L. Inactivation of two mouse alkaline phosphatase genes and establishment of a model of infantile hypophosphatasia. *Dev Dyn* **1997**, *208*, 432-446, doi:10.1002/(SICI)1097-0177(199703)208:3<432::AID-AJA13>3.0.CO;2-1.

12. Narisawa, S.; Huang, L.; Iwasaki, A.; Hasegawa, H.; Alpers, D.H.; Millan, J.L. Accelerated fat absorption in intestinal alkaline phosphatase knockout mice. *Mol Cell Biol* **2003**, *23*, 7525-7530, doi:10.1128/mcb.23.21.7525-7530.2003.

13. Nagata, K.; Kiryu-Seo, S.; Maeda, M.; Yoshida, K.; Morita, T.; Kiyama, H. Damage-induced neuronal endopeptidase is critical for presynaptic formation of neuromuscular junctions. *J Neurosci* **2010**, *30*, 6954-6962, doi:10.1523/JNEUROSCI.4521-09.2010.

14. Takahashi, M.; Kubo, T.; Mizoguchi, A.; Carlson, C.G.; Endo, K.; Ohnishi, K. Spontaneous muscle action potentials fail to develop without fetal-type acetylcholine receptors. *EMBO Rep* **2002**, *3*, 674-681, doi:10.1093/embo-reports/kvf128.

15. Morita, M.; Ler, L.W.; Fabian, M.R.; Siddiqui, N.; Mullin, M.; Henderson, V.C.; Alain, T.; Fonseca, B.D.; Karashchuk, G.; Bennett, C.F., et al. A novel 4EHP-GIGYF2 translational repressor complex is essential for mammalian development. *Mol Cell Biol* **2012**, *32*, 3585-3593, doi:10.1128/MCB.00455-12.

16. Nair, K.S.; Hmani-Aifa, M.; Ali, Z.; Kearney, A.L.; Ben Salem, S.; Macalinao, D.G.; Cosma, I.M.; Bouassida, W.; Hakim, B.; Benzina, Z., et al. Alteration of the serine protease PRSS56 causes angle-closure glaucoma in mice and posterior microphthalmia in humans and mice. *Nat Genet* **2011**, *43*, 579-584, doi:10.1038/ng.813.

17. Tang, T.; Li, L.; Tang, J.; Li, Y.; Lin, W.Y.; Martin, F.; Grant, D.; Solloway, M.; Parker, L.; Ye, W., et al. A mouse knockout library for secreted and transmembrane proteins. *Nat Biotechnol* **2010**, *28*, 749-755, doi:10.1038/nbt.1644.

18. Sahin, M.; Greer, P.L.; Lin, M.Z.; Poucher, H.; Eberhart, J.; Schmidt, S.; Wright, T.M.; Shamah, S.M.; O'Connell, S.; Cowan, C.W., et al. Eph-dependent tyrosine phosphorylation of ephexin1 modulates growth cone collapse. *Neuron* **2005**, *46*, 191-204, doi:10.1016/j.neuron.2005.01.030.

19. Nguyen, N.Y.; Maxwell, M.J.; Ooms, L.M.; Davies, E.M.; Hilton, A.A.; Collinge, J.E.; Hilton, D.J.; Kile, B.T.; Mitchell, C.A.; Hibbs, M.L., et al. An ENU-induced mouse mutant of SHIP1 reveals a critical role of the stem cell isoform for suppression of macrophage activation. *Blood* **2011**, *117*, 5362-5371, doi:10.1182/blood-2011-01-331041.

20. Cadwell, K.; Liu, J.Y.; Brown, S.L.; Miyoshi, H.; Loh, J.; Lennerz, J.K.; Kishi, C.; Kc, W.; Carrero, J.A.; Hunt, S., et al. A key role for autophagy and the autophagy gene Atg16l1 in mouse and human intestinal Paneth cells. *Nature* **2008**, *456*, 259-263, doi:10.1038/nature07416.

21. Xu, J.; Dodd, R.L.; Makino, C.L.; Simon, M.I.; Baylor, D.A.; Chen, J. Prolonged photoresponses in transgenic mouse rods lacking arrestin. *Nature* **1997**, *389*, 505-509, doi:10.1038/39068.

22. Crotty, T.; Cai, J.; Sakane, F.; Taketomi, A.; Prescott, S.M.; Topham, M.K. Diacylglycerol kinase delta regulates protein kinase C and epidermal growth factor receptor signaling. *Proc Natl Acad Sci U S A* **2006**, *103*, 15485-15490, doi:10.1073/pnas.0604104103.

23. Dhaka, A.; Murray, A.N.; Mathur, J.; Earley, T.J.; Petrus, M.J.; Patapoutian, A. TRPM8 is required for cold sensation in mice. *Neuron* **2007**, *54*, 371-378, doi:10.1016/j.neuron.2007.02.024.

24. Ward-Bailey, P.F.; Harris, B.S.; Donahue, L.R.; Bronson, R.; Johnson, K.R. Long bone Abnormality (lbab): A New Spontaneous Mutation Causing Small Size and Skeletal Abnormalities on Chromosome 1 in the Mouse. The Jackson Laboratory: Bar Harbor: ME, USA, 2002.

25. Consortium, I.K.M. MGI download of modified allele data from IKMC and creation of new knockout alleles. Database Download: 2014.
